# Traumatic injury is associated with reduced deoxyribonuclease activity and dysregulation of the actin scavenging system

**DOI:** 10.1093/burnst/tkab001

**Published:** 2021-04-01

**Authors:** Jon Hazeldine, Robert J Dinsdale, David N Naumann, Animesh Acharjee, Jonathan R B Bishop, Janet M Lord, Paul Harrison

**Affiliations:** 1 Institute of Inflammation and Ageing, University of Birmingham, Edgbaston, Birmingham, West Midlands, B15 2TT, United Kingdom; 2 National Institute for Health Research Surgical Reconstruction and Microbiology Research Centre, Heritage Building, Queen Elizabeth Hospital Birmingham, Mindelsohn Way, Edgbaston, Birmingham, West Midlands, B15 2TH, United Kingdom; 3 Scar Free Foundation Birmingham Centre for Burns Research, University Hospital Birmingham Foundation Trust, Queen Elizabeth Hospital Birmingham, Mindelsohn Way, Edgbaston, Birmingham, West Midlands, B15 2TH, United Kingdom; 4 Academic Department of Military Surgery and Trauma, Royal Centre for Defence Medicine, Queen Elizabeth Hospital Birmingham, Mindelsohn Way, Edgbaston, Birmingham, West Midlands, B15 2TH, United Kingdom; 5 Institute of Cancer and Genomic Sciences, Centre for Computational Biology, University of Birmingham, Edgbaston, Birmingham, West Midlands, B15 2TT, United Kingdom

**Keywords:** Cell-free DNA, Deoxyribonuclease, Extracellular actin scavenging system, Pre-hospital, Trauma

## Abstract

**Background:**

Traumatic injury is associated with increased concentrations of cell-free DNA (cfDNA) in the circulation, which contribute to post-injury complications. The endonuclease deoxyribonuclease 1 (DNase-1) is responsible for removing 90% of circulating cfDNA. Recently, DNase activity was reported to be significantly reduced following major non-traumatic brain injury (TBI), but the processes responsible were not investigated. Moreover, it is not known how quickly following injury DNase activity is reduced and whether this also occurs after TBI.

**Methods:**

At 3 post-injury time points (≤1, 4–12 and 48–72 hours), blood samples were obtained from 155 adult trauma patients that had sustained an isolated TBI (n = 21), TBI with accompanying extracranial injury (TBI^+^) (n = 53) or an extracranial injury only (ECI) (n = 81). In addition to measuring cfDNA levels and the activity and expression of DNase, circulating concentrations of monomeric globular action (G-actin), an inhibitor of DNase-1, and the actin scavenging proteins gelsolin (GSN) and vitamin D binding protein (VDBP) were determined and values compared to a cohort of healthy controls.

**Results:**

Significantly elevated concentrations of plasma cfDNA were seen in TBI, TBI^+^ and ECI patients at all study time points when compared to healthy controls. cfDNA levels were significantly higher at ≤1 hour post-injury in ECI patients who subsequently developed multiple organ dysfunction syndrome when compared to those who did not. Plasma DNase-1 protein was significantly elevated in all patient groups at all sampling time points. In contrast, DNase enzyme activity was significantly reduced, with this impaired function evident in TBI^+^ patients within minutes of injury. Circulating concentrations of G-actin were elevated in all patient cohorts in the immediate aftermath of injury and this was accompanied by a significant reduction in the levels of GSN and VDBP.

**Conclusions:**

The post-traumatic increase in circulating cfDNA that occurs following extracranial trauma and TBI is accompanied by reduced DNase activity. We propose that, secondary to reduced GSN and VDBP levels, elevated circulating concentrations of G-actin underlie the post-injury reduction in DNase activity. Reducing circulating cfDNA levels via therapeutic restoration of DNase-1 activity may improve clinical outcomes post-injury.

Highlights

- Via the analysis of blood samples acquired from major trauma patients within 1 hour of injury, this study is the first to examine the immediate impact of trauma on the activity of circulating deoxyribonuclease.
- Following isolated traumatic brain injury, we detected a very rapid post-injury reduction in deoxyribonuclease activity that was accompanied by elevated concentrations of circulating cell-free DNA.
- The post-injury impairment in deoxyribonuclease activity occurred secondary to elevated levels of monomeric globular actin, an inhibitor of deoxyribonuclease, and reduced concentrations of the actin scavengers gelsolin and vitamin D binding protein.
- Significantly higher levels of cell-free DNA were detected in plasma samples acquired within 1 hour of injury from extracranial trauma patients who subsequently developed multiple organ dysfunction syndrome when compared to those with a non-eventful outcome.
- Reducing circulating cell-free DNA levels via therapeutic restoration of deoxyribonuclease activity may improve clinical outcomes post-injury.

## Background

Traumatic injury results in raised levels of cell-free DNA (cfDNA) in the circulation. Released from damaged/necrotic tissue, and as a by-product of immune cell activation [1–3], significantly increased concentrations of mitochondrial-derived DNA (mtDNA) [3–8] and nuclear-derived DNA (nDNA) [4–6, 9–14] have been measured in blood samples acquired from trauma patients with different mechanisms of injury, such as traumatic brain injury (TBI) [6, 9, 10, 13, 15], blunt trauma [4, 5, 12] and burns [11, 16, 17]. Attributed, in part, to its pro-thrombotic and cytotoxic nature [18–22], elevated cfDNA levels post trauma have been linked to a number of adverse clinical outcomes, which include the development of acute respiratory distress syndrome (ARDS) [23], multiple organ dysfunction syndrome (MODS) [8, 14, 24], multiple organ failure [14] and death [4, 7, 9, 10, 12, 13]. However, in spite of its potential impact on patient outcomes, the mechanisms behind the post-trauma elevation in circulating cfDNA are still uncertain.

Prospective observational cohort studies of trauma patients have shown that, relative to healthy controls (HCs), circulating cfDNA concentrations remain significantly elevated in the days, weeks and months following injury [5, 11, 13, 14, 25, 26]. With the half-life of DNA in circulation estimated to be less than 2 hours [27, 28], the persistent elevation in cfDNA levels post-injury could be a consequence of sustained production, triggered by secondary events such as surgical intervention [5, 29, 30] and/or its impaired breakdown and clearance. As the predominant endonuclease in bodily fluids, deoxyribonuclease 1 (DNase-1) is responsible for the breakdown of 90% of circulating cfDNA [31]. With the exception of a significant increase on the day of injury, major trauma has no effect on the circulating concentration of DNase-1 protein [25]. However, a persistent post-injury impairment in DNase activity has been described. Analysing plasma samples from 103 trauma patients with major extracranial injuries, McIlroy *et al.* reported elevated mtDNA levels and significantly reduced DNase activity in the circulation [26]. Although the first study to report a trauma-induced reduction in the activity of this endonuclease, the authors did not investigate the mechanism(s) involved, nor did they determine whether this reduction in DNase activity occurred in cohorts of trauma patients with different injuries, such as TBI.

DNase-1 activity is negatively regulated by actin, a cytoskeletal protein that exists in 2 forms: monomeric globular actin (G-actin) and filamentous actin (F-actin). Released into the circulation as a result of cell turnover, damage or death, G-actin forms a stable 1:1 stoichiometric complex with DNase-1, inhibiting its nuclease activity [32–34]. Facilitating the removal of actin from the circulation is the extracellular actin scavenger system (EASS), which comprises 2 plasma proteins: vitamin D binding protein (VDBP) and gelsolin (GSN). Mechanistically, GSN binds to and drives the depolymerization of F-actin, generating monomeric G-actin subunits that are bound by VDBP to form complexes that are cleared by the liver, spleen and kidneys [35–37]. The importance of the EASS for the breakdown of cfDNA is demonstrated by reports that GSN-mediated dissociation of actin from DNase-1 enhances its endonuclease activity [38].

Traumatic injury is associated with dysregulation of the EASS. Demonstrating the immediate post-injury release of actin into circulation, increased frequencies of actin–VDBP complexes have been detected in hospital admission blood samples of trauma patients [39], who also present with immediate and sustained reductions in circulating concentrations of VDBP [39, 40] and GSN [41–44]. However, to date, no single study has measured cfDNA, DNase activity/expression and components of the EASS in trauma patients. Moreover, it is currently unknown whether DNase activity is impaired in the immediate aftermath of trauma, and whether injuries other than extracranial trauma result in reduced activity of this endonuclease.

The aim of this prospective observational cohort study was to examine cfDNA levels, the expression and activity of DNase and the circulating concentrations of GSN and VDBP in blood samples acquired during the immediate (≤1 hour) and acute (4–72 hours) post-injury phase from patients that had sustained an isolated TBI, TBI with accompanying extracranial injury (TBI^+^) or an extracranial injury (ECI) only.

## Methods

### Study design

This article reports data acquired from subjects enrolled in the Brain Biomarkers after Trauma Study between 15 May 2014 and 26 August 2018. This study is an ongoing prospective longitudinal observational study of adult trauma patients conducted at a single major trauma centre in the UK (University Hospitals Birmingham National Health Service (NHS) Foundation Trust, Birmingham). Ethical approval for the study was granted by the North Wales Research Ethics Committee—West (REC reference: 13/WA/0399, protocol number: RG_13–164). Due to the nature of their injuries, patients were unlikely to be able to provide informed consent to enroll in the study. As such, patient recruitment into the study was performed under the guidance of the Mental Health Capacity Act for research in emergency situations, in accordance with the Declaration of Helsinki. Details on patient selection, study enrolment and the consenting procedure for patients who lacked capacity have been described previously [2]. A total of 75 adults (mean age, 41 years; range, 18–78 years; 47 male) were enrolled as a cohort of HCs (Table 1).

### Clinical outcomes

The patient outcomes assessed in this study were MODS (defined as a Sequential Organ Failure Assessment (SOFA) score of ≥6 on 2 or more consecutive days, at least 48 hours post admission) and hospital-free and intensive care unit (ICU)-free days, which were calculated as 30 minus the number of days the patient stayed in hospital and ICU, respectively. Patients who died in the hospital or ICU setting within 30 days of admission were assigned a score of 0.

### Clinical data collection

Patient and injury details were collected prospectively from contemporaneous electronic and physical medical records for included patients. These included age, gender, mechanism of injury, severity of injury (Injury Severity Score (ISS) and New ISS (NISS)), and Glasgow Coma Scale (GCS). Data required for SOFA (and MODS) assessment included ventilation status, PaO_2_, FiO_2_, mean arterial pressure and bilirubin, platelet and creatinine concentrations. The assigned SOFA scores for each day were taken as the highest score for that day.

### Blood sampling

The blood samples were collected in BD Vacutainers® (BD Biosciences, UK) containing z-serum clotting activator or a 1/10 volume of 3.2% trisodium citrate at 3 post-injury time points pre-hospital (≤1-hour), 4–12 and 48–72 hours. For samples acquired in the pre-hospital setting, Vacutainers were stored at room temperature (RT) during transportation to hospital where, upon arrival, they were stored at 4°C and collected for analysis within 1 hour by a single laboratory researcher on a 24-hour basis.

### Injury classification

Patients were divided into groups within the study based on their initial clinical and radiological assessments. In brief, radiological assessment consisted of a non-contrast head CT followed by a contrast-enhanced CT (with biphasic injection of contrast) from the circle of Willis to the level of the greater trochanters. These results, along with secondary and tertiary patient examinations, were used by a minimum of 2 independent clinicians to determine whether patients were classified as having suffered an isolated TBI, a TBI with accompanying ECI or an ECI only.

### Preparation of platelet-free plasma (PFP) and serum

PFP was generated by a two-step centrifugation process. Citrate anticoagulated blood was centrifuged at 2000 × *g* for 20 minutes at 4°C, after which the top two-thirds of platelet-poor plasma was carefully removed. Following centrifugation of platelet-poor plasma at 13,000 × *g* for 2 minutes at 4°C, PFP was collected and stored at −80°C until analysed. To prepare serum, blood samples collected into Vacutainers containing z-serum clotting activator were incubated at RT for 30 minutes before centrifugation at 1620 × *g* for 10 minutes at RT. Serum aliquots were stored at −80°C prior to analysis.

### Fluorometric analysis of cfDNA

cfDNA levels in PFP were measured using a fluorometric-based assay. A sample of 10 μL of PFP was incubated for 10 minutes in the dark at RT with 1 μM SYTOX green dye (Life Technologies, UK), after which fluorescence was measured using a BioTek Synergy 2 fluorometric plate reader (NorthStar Scientific Ltd, UK). with excitation and emission set at 485 nm and 528 nm, respectively. For quantification, a λ-DNA standard curve (Fisher Scientific, UK) was used. PFP samples were run in duplicate, with cfDNA concentrations calculated from the average value via extrapolation from the standard curve.

### DNase activity

Neutrophil extracellular traps (NETs) served as the DNA substrate for our studies of DNase activity in serum [45]. To generate NETs, neutrophils (5 × 10^5^) isolated from ethylenediaminetetraacetic acid anticoagulated blood by Percoll density gradient centrifugation were dispensed into wells of a 96-well flat-bottomed plate (BD Biosciences, UK) and stimulated for 3 hours (37°C/5% CO_2_) with 25 nM phorbol 12-myristate 13-acetate (Sigma-Aldrich, UK). Post stimulation, NETs were incubated for 6 hours (37°C/5% CO_2_) with 5% patient sera diluted in Hank’s balanced salt solution supplemented with calcium and magnesium (Gibco, Life Technologies, UK), or in Hank’s balanced salt solution supplemented with calcium and magnesium alone. Following a 30-minute fixation with 5% paraformaldehyde (Sigma-Aldrich, UK) (37°C/5% CO_2_), samples were washed 3 times with phosphate-buffered saline (Sigma-Aldrich, UK) prior to a 10-minute incubation at RT in the dark with 1 μM SYTOX green dye. Fluorescence was measured using a BioTek Synergy 2 fluorometric plate reader with excitation and emission set at 485 nm and 528 nm, respectively. NET degradation by 5% serum pooled from 10 HCs was used to define 100% DNase activity.

### Enzyme-linked immunosorbent assay (ELISA)

Performed in accordance with the instructions of the manufacturers, serum concentrations of GSN (LifeSpan BioSciences Inc., UK) and G-actin (MyBioSource, USA) were quantified using ELISAs. PFP samples were analysed to determine the concentrations of circulating DNase-1 (LifeSpan BioSciences Inc, UK) and VDBP (Abcam, UK).

### Lactate dehydrogenase (LDH) activity assay

LDH activity in 5-μL serum samples was quantified using the LDH activity assay kit (Sigma-Aldrich, UK) according to the manufacturer’s instructions.

### Data analysis

Statistical analyses were performed using GraphPad Prism software version 5 (GraphPad Software Ltd, USA) and R (v3.6.1, https://www.R-project.org). Data distributions were examined using the Shapiro–Wilk normality test. For the comparison of HC and patient data, either the Mann–Whitney *U* test or the unpaired *t*-test with a Bonferroni correction for multiple comparisons was performed. Differences in cfDNA concentrations at each sampling time point between patients who did or did not develop MODS were examined using the Mann–Whitney *U* test. Spearman correlations were calculated to examine the relationship between cfDNA concentration and hospital- or ICU-free days. Differences in cfDNA concentrations and LDH activity between patient groups were compared using a two-way analysis of variance. Area under the receiver operating characteristic (AUROC) analysis was performed to estimate the predictability of MODS development based on the pre-hospital concentrations of cfDNA. The scaled (*Z*-transformed) values were considered for sensitivity, specificity and corresponding AUROC values. For Mann–Whitney *U* tests and unpaired *t-*tests, where a Bonferroni correction for multiple comparisons was performed, statistical significance was set at an adjusted *p* value of <0.016. For all other analyses, statistical significance was accepted at *p* < 0.05. Data are presented as Tukey-style box and whisker plots, where the line inside the box represents the median value and outliers are indicated by individual dots.

## Results

### Patient characteristics

Of the 155 trauma patients included in this study, 21, 53 and 81 were classified as having sustained an isolated TBI, TBI^+^ or ECI, respectively (Table 1). Isolated TBI patients, from whom pre-hospital blood samples were acquired within a mean time of 44 minutes (range, 26–60 minutes) post-injury, were a predominantly male cohort (86%) with severe head trauma, evidenced by a mean GCS score of 7 (range, 3–15) and an ISS of 25 (range, 9–45) (Table 1). The primary mechanism of injury was a fall from height, which accounted for 43% of injuries sustained (Table 1). For the TBI^+^ cohort, of which 81% were male, pre-hospital blood samples were acquired within a mean time of 42 minutes (range, 13–60 minutes) post-injury (Table 1). The mean ISS and NISS were 34 (range, 9–66) and 49 (range, 12–75), respectively, with road traffic collisions the predominant mechanism of injury (Table 1). Road traffic collisions and assault/penetrating injuries were the primary mechanisms of injury amongst our cohort of ECI patients, of whom 90% were male (Table 1). ECI patients presented with mean ISS and NISS scores of 18 (range, 9–75) and 25 (range, 9–75), respectively, with pre-hospital blood samples obtained within a mean time of 40 minutes (range, 18–60 minutes) post-injury (Table 1).

**Table 1:** Patient Demographics

**Variables**	**HCs (n = 75)**	**TBI (n = 21)**	**TBI** ^**+**^ **(n = 53)**	**ECI (n = 81)**
Age, years	41 (18-78)	41 (19-81)	42 (18-87)	40 (18-95)
Male, n (%)	47 (63)	18 (86)	43 (81)	73 (90)
Time to pre-hospital	-	44 (26-60)	42 (13-60)	40 (18-60)
sample, minutes ISS	-	25 (9-45)	34 (9-66)	18 (9-75)
NISS	-	48 (19-75)	49 (12-75)	25 (9-75)
GCS	-	7 (3-15)	-	-
Mechanism of Injury				
Fall, n (%)	-	9 (43)	10 (19)	10 (12)
A/P, n (%)	-	3 (14)	0	33 (41)
Blunt, n (%)	-	1 (5)	0	5 (6)
RTC, n (%)	-	8 (38)	43 (81)	33 (41)
Outcomes				
ICU-free days	-	21 (0-30)	15 (0-30)	25 (0-30)
Hospital free days	-	12 (0-29)	4 (0-26)	15 (0-29)
MODS, n (%)	-	11 (52)	35 (66)	17 (21)
Mortality, n (%)	-	4 (19)	15 (28)	5 (6)

Data are expressed as mean (range) unless otherwise stated

For TBI, TBI^+^ and ECI patient groups, the number of data points for the clinical variables ISS and NISS are 19, 52 and 73 respectively

ICU-free days and hospital-free days were calculated as 30 minus the number of days the patient stayed in the respective settings. Patients who died in ICU within 30 days of hospital admission were assigned a hospital and ICU-free day score of 0

*HCs* healthy controls, *TBI* traumatic brain injury, *TBI^+^* TBI with accompanying extracranial injury, *ECI* extracranial injury, *A/P* assault/penetrating, *GCS* Glasgow coma scale, *ICU* intensive care unit, *ISS* Injury Severity Score, *MODS* multiple organ dysfunction syndrome, *NISS* new injury severity score, *RTC* road traffic collision

### Increased plasma cfDNA levels after traumatic injury are associated with poor patient outcomes

**Figure 1.**
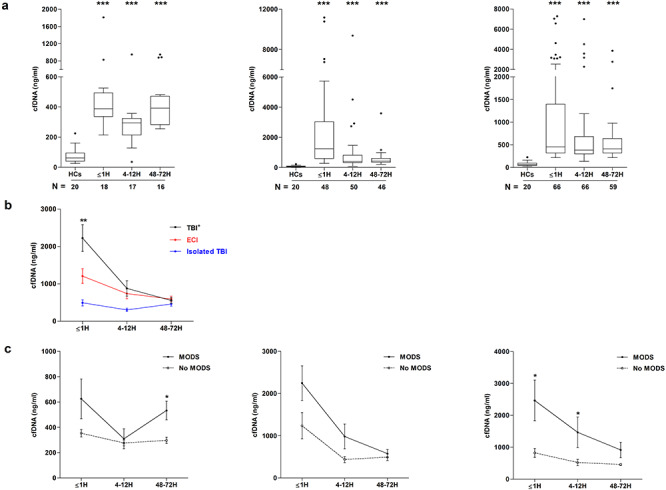
Trauma-induced changes in plasma cell-free DNA (cfDNA) levels and their relationship with patient outcome. **(a)** Comparison of cfDNA concentrations in plasma samples acquired from healthy controls (HCs) and patients that had sustained an isolated traumatic brain injury (TBI) (**left panel**), TBI with an accompanying extracranial injury (TBI^+^) (**middle panel**) or an extracranial injury (ECI) only (**right panel**). Patient samples were acquired at 3 post-injury time points (≤1 hour, 4–12 hours and 48–72 hours), with the number of samples analysed indicated below each time point. ^*^^*^^*^p < 0.0001 *vs* HCs. **(b)** Comparison of cfDNA concentrations in plasma samples from TBI, TBI^+^ and ECI patients at 3 post-injury time points. Number of samples analysed match those reported in (a). ^*^^*^p < 0.001 *vs* ECI and isolated TBI. **(c)** Comparison of cfDNA concentrations in plasma samples obtained from TBI, TBI^+^ and ECI patients who did or did not develop multiple organ dysfunction syndrome (MODS) during their hospital stay (TBI: MODS ≤1 hour (H) n = 9, 4–12H n = 10, 48–72H n = 11; no MODS ≤1H n = 7, 4–12H n = 5, 48–72H n = 5; TBI^+^: MODS ≤1H n = 32, 4–12H n = 34, 48–72H n = 35; no MODS ≤1H n = 11, 4–12H n = 13, 48–72H n = 11; ECI: MODS ≤1H n = 16, 4–12H n = 15, 48–72H n = 17; no MODS ≤1H n = 43, 4–12H n = 43, 48–72H n = 41). ^*^p < 0.05 *vs* no MODS. *H* hour

At all post-injury time points, significantly higher concentrations of cfDNA were measured in plasma from isolated TBI, TBI^+^ and ECI patients when compared to HCs (Figure 1a). A comparison of cfDNA concentrations between patient groups revealed significantly higher levels of cfDNA in plasma samples acquired within 1 hour of injury from TBI^+^ patients when compared to individuals that had sustained either an isolated TBI or ECI (Figure 1b). For TBI^+^ and ECI patients, no relationship was found between the concentration of cfDNA in samples obtained ≤1 hour post-injury and either ISS (TBI^+^, *r*(*n* = 47) = 0.184, *p* = 0.21; ECI, *r*(*n* = 60) = 0.07, *p* = 0.56) or NISS (TBI^+^, *r*(*n* = 47) = −0.004, *p* = 0.976; ECI, *r*(*n* = 60) = 0.126, *p* = 0.33). Similarly, in our cohort of isolated TBI patients, cfDNA concentrations in plasma samples obtained ≤1 hour post-injury did not correlate with GCS scores (*r*(*n* = 18) = −0.338, *p* = 0.17).

To investigate whether cfDNA levels were associated with clinical outcomes, we first compared the concentration of cfDNA in plasma samples between patients who did or did not develop MODS during their hospital stay. In our isolated TBI cohort, plasma cfDNA levels were higher in samples obtained ≤1 hour post-injury from patients who developed MODS, although this was not statistically significant (*p* = 0.054) (Figure 1c). By 48–72 hours post-injury, cfDNA concentrations were significantly increased in isolated TBI patients who subsequently developed MODS (Figure 1c). At no sampling time point were cfDNA levels different between TBI^+^ patients who did or did not develop MODS (Figure 1c). In our ECI cohort, significantly elevated cfDNA concentrations were measured in the ≤1 hour and 4–12 hour post-injury plasma samples of subjects who developed MODS (Figure 1c). Focusing on ECI patients, who represented our largest cohort, we examined the ability of cfDNA to discriminate between the MODS and no MODS cohorts using AUROC analysis. We found that cfDNA concentrations in samples acquired within an hour of injury moderately discriminated between the 2 groups (AUROC 0.729, 95% CI 0.564–0.866; sensitivity 0.73, 95% CI 0.63–0.85; specificity 0.62, 95% CI 0.51–0.70).

An examination of whether cfDNA levels were associated with the number of hospital or ICU-free days revealed a significant negative relationship between cfDNA and both outcome measures at all sampling time points for patients that had sustained an ECI (Table 2). Within our isolated TBI cohort, cfDNA concentrations in ≤1 hour post-injury plasma samples were negatively associated with both ICU- and hospital-free days (Table 2). For TBI^+^ patients, a significant negative relationship was observed between cfDNA concentrations at the 4–12 hour sampling time point and the number of hospital-free days (Table 2).

**Table 2:** Correlations between cfDNA concentrations at pre-hospital (≤1H), 4-12H and 48-72H post-injury time-points and length of stay outcomes

	**Hospital-free days**			**ICU-free days**		
	**≤1H**	**4-12H**	**48-72H**	**≤1H**	**4-12H**	**48-72H**
TBI	**-**0.522 (-0.800– -0.058)	-0.013 (-0.503–0.481)	-0.378 (-0.743–0.160)	-0.490 (-0.784– -0.015)	-0.118 (-0.577 – 0.397)	-0.242 (-0.668–0.302)
TBI^+^	-0.131 (-0.407–0.167)	-0.319 (-0.555– -0.037)	-0.198 (-0.469–0.105)	-0.056 (-0.342–0.239)	-0.248 (-0.498–0.041)	-0.128 (-0.410–0.177)
ECI	-0.470 (-0.643– -0.250)	-0.289 (-0.502– -0.043)	-0.279 (-0.505– -0.017)	-0.482 (-0.652– -0.265)	-0.356 (-0.555– -0.117)	-0.342 (-0.555– -0.087)

Data are presented as r, with 95% confidence intervals in parentheses

Significant associations according to Spearman’s rank correlation coefficient are indicated in bold font.

Number of pairs for hospital/ICU-free days and cfDNA correlations: ≤1H, TBI (n=18), TBI^+^ (n=48), ECI (n=66); 4-12H, TBI (n=17), TBI^+^ (n=49), ECI (n=66); 48-72H, TBI (n=16), TBI^+^ (n=46), ECI (n=59).

*cfDNA* cell-free DNA, *ECI* extracranial injury, *ICU* intensive care unit, *TBI* traumatic brain injury, *TBI^+^* TBI with accompanying extracranial injury, *H* hour

### Effect of traumatic injury on the activity and expression of circulating DNase

**Figure 2.**
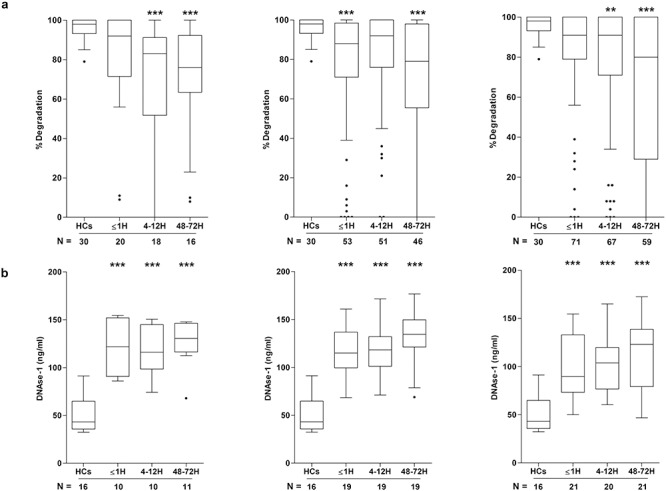
Effect of traumatic injury on deoxyribonuclease (DNase) activity and antigen levels. Comparison of DNase activity **(a)** and antigen levels **(b)** in peripheral blood samples acquired from healthy controls (HCs) and patients that had sustained an isolated traumatic brain injury (TBI) (**left panel**), TBI with an accompanying extracranial injury (**middle panel**) or an extracranial injury only (**right panel**). Patient samples were acquired at 3 post-injury time points (≤1 hour (H), 4–12 hours and 48–72 hours) and the number of samples analysed is indicated below each time point. ^*^^*^p < 0.01, ^*^^*^^*^p < 0.005 *vs* HCs. *H* hour

Reduced breakdown is a potential explanation for the persistent elevation in circulating cfDNA post trauma. To examine this possibility, we measured DNase activity. Compared to HCs, isolated TBI patients presented with significantly reduced DNase activity at the 4–12 and 48–72 hour post-injury time points (Figure 2a). In our TBI^+^ cohort, a significant impairment in DNase activity was detected in both pre-hospital and 48–72 hour post-injury samples (Figure 2a). At the 4–12 hour and 48–72 hour post-injury time points, ECI patients presented with significantly reduced DNase activity (Figure 2a). DNase activity was also lower in samples acquired from ECI patients within 1 hour of injury, although this was not statistically significant (*p* = 0.016) (Figure 2a).

To investigate whether the post-trauma impairment in DNase activity was a consequence of reduced DNase protein levels, we quantified DNase-1 by ELISA. As shown in Figure 2b, compared to HCs, significantly elevated levels of DNase-1 were measured in blood samples acquired ≤1, 4–12 and 48–72 hours post-injury from all 3 patient groups.

### Serum LDH activity and G-actin concentration are increased following traumatic injury

**Figure 3.**
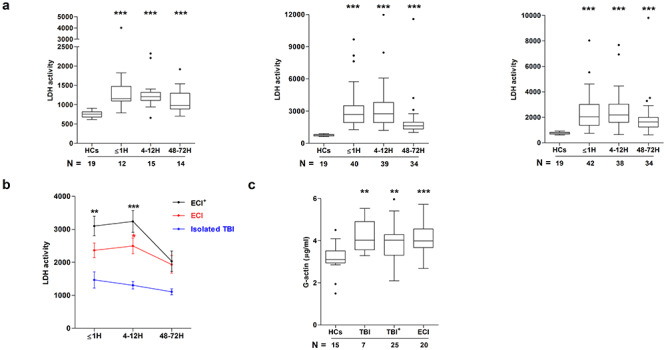
Trauma-induced elevation in circulating lactate dehydrogenase (LDH) activity and globular actin (G-actin) concentration. **(a)** Comparison of LDH activity in serum samples obtained from healthy controls (HCs) and patients that had sustained an isolated traumatic brain injury (TBI) (**left panel**), TBI with an accompanying extracranial injury (TBI^+^) (**middle panel**) or an extracranial injury (ECI) only (**right panel**). Patient samples were acquired at 3 post-injury time points (≤1 hour (H), 4–12 hours and 48–72 hours) and the number of samples analysed is indicated below each time point. LDH activity is presented as milliunits/ml. ^*^^*^^*^p < 0.005 *vs* HCs. **(b)** Comparison of LDH activity in serum samples from TBI, TBI^+^ and ECI patients at 3 post-injury time points. Number of samples analysed match those reported in **(a)**. ^*^p < 0.05, ^*^^*^p < 0.01, ^^*^^*^^p < 0.001 *vs* Isolated TBI. **(c)** Comparison of the concentration of G-actin in pre-hospital blood samples acquired from TBI, TBI^+^ and ECI patients within 1 hour of injury and HCs. The number of samples analysed are indicated below each time point. ^*^^*^p < 0.01, ^*^^*^^*^p < 0.005 *vs* HCs. *H* hour

At all sampling time points, serum LDH activity, a marker of cell and tissue damage, was significantly higher in each of our patient groups when compared to HCs (Figure 3a). In serum samples acquired from TBI^+^ patients ≤1 hour and 4–12 hours post-injury, and from ECI patients 4–12 hours post-injury, LDH activity was significantly greater than that recorded in samples obtained at the corresponding time points from isolated TBI patients (Figure 3b). For both TBI^+^ and ECI patients, LDH activity and cfDNA levels in samples acquired within 1 hour of injury were positively associated (TBI^+^, *r*(*n* = 35) = 0.696, *p* = <0.0001; ECI, *r*(*n* = 29) = 0.524, *p* = 0.003).

Released from injured tissue, G-actin inhibits the nuclease activity of DNase-1 [32–34]. Focusing on samples collected in the immediate aftermath of injury, we detected a significant increase in the concentration of G-actin in pre-hospital blood samples acquired from all 3 patient groups when compared to HCs (Figure 3c).

### Impact of trauma on the circulating levels of GSN and VDBP

**Figure 4.**
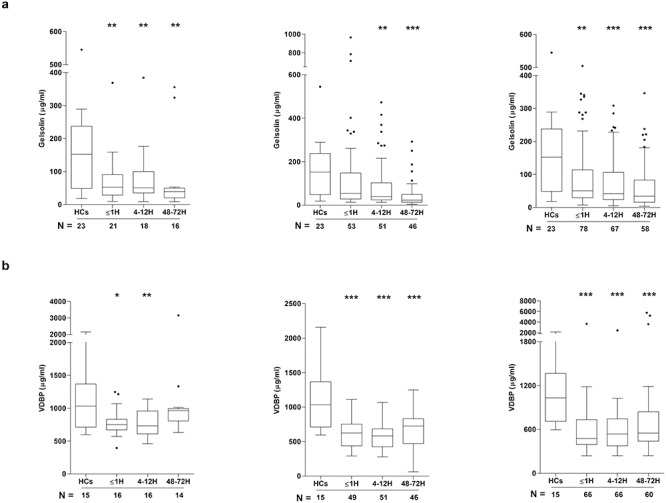
Effect of traumatic injury on the circulating extracellular actin scavenging system. Comparison of the concentrations of gelsolin **(a)** and vitamin D binding protein (VDBP) **(b)** in peripheral blood samples acquired from healthy controls (HCs) and patients that had sustained an isolated traumatic brain injury (TBI) (**left panel**), TBI with an accompanying extracranial injury (**middle panel**) or an extracranial injury only (**right panel**). Patient samples were acquired at 3 post-injury time points (≤1 hour (H), 4–12 hours and 48–72 hours) and the number of samples analysed is indicated below each time point. ^*^p < 0.05, ^*^^*^p < 0.01, ^*^^*^^*^p < 0.005 *vs* HCs. *H* hour

As part of the EASS, the plasma protein GSN drives the depolymerization of F-actin [35]. At all 3 sampling time points, GSN concentrations, relative to HCs, were significantly reduced in samples acquired from patients that had sustained either an isolated TBI or ECI (Figure 4a). In the TBI^+^ cohort, a significant trauma-induced reduction in GSN levels was detected in samples obtained 4–12 hours and 48–72 hours post-injury (Figure 4a). Subunits of monomeric G-actin are bound by VDBP to form complexes that are cleared by the liver, spleen and kidneys [36, 37]. At all sampling time points, concentrations of VDBP were significantly reduced in plasma samples from ECI and TBI^+^ patients when compared to the levels recorded in samples from HCs (Figure 4b). In isolated TBI patients, VDBP levels were significantly lower ≤1 hour and 4–12 hours post-injury (Figure 4b).

## Discussion

In this prospective observational cohort study of 155 trauma patients, from whom blood samples were acquired during the ultra-early (≤1 hour) and acute (4–72 hours) post-injury phase, we have shown for the first time that major trauma results in an immediate impairment in DNase activity. Accompanied by significantly elevated circulating concentrations of cfDNA, this reduction in DNase activity was observed in patients that had sustained extracranial and brain injuries. Identifying a potential underlying mechanism for this trauma-associated decline in DNase activity, circulating concentrations of G-actin, an inhibitor of DNase-1, were significantly increased within minutes of injury, whilst plasma levels of the actin scavengers GSN and VDBP were significantly reduced. With our data suggesting that elevated circulating concentrations of cfDNA are associated with the development of MODS in ECI and isolated TBI patients, therapeutic restoration of DNase activity may represent a strategy by which to reduce poor clinical outcome after a broad range of injuries.

In agreement with some, but not all, previous studies that have examined the relationship between injury burden and circulating cfDNA levels [46], we found no association between the concentration of cfDNA in pre-hospital plasma samples and injury severity. However, we did find that elevated cfDNA levels during the ultra-early (≤1 hour) and acute (4–72 hours) post-injury phases were associated with poorer clinical outcome. Mirroring the findings of Shaked *et al.* [15], we observed, in all 3 of our patient groups, a significant negative association between plasma cfDNA concentrations and the number of hospital- and/or ICU-free days. However, only in our cohort of ECI patients was this relationship between raised cfDNA levels and a longer hospital/ICU length of stay (LOS) evident at all 3 sampling time points, suggesting that cfDNA may be a better indicator of outcome in patients with extracranial injuries when compared to subjects with head trauma. Recently, it has been proposed that the release of cfDNA post trauma is more likely to be representative of injury burden and the degree of tissue damage sustained by a patient rather than a marker of early physiological derangement [47]. In line with this theory, Ren and colleagues found that the percentage of plasma samples positive for cfDNA was significantly higher in a cohort of trauma patients with penetrative injuries when compared to individuals that had sustained blunt trauma [48]. If, as these data suggest, soft tissue is the predominant source of cfDNA, and cfDNA is a marker of overall tissue damage and injury burden [47], then the higher incidence of assault/penetrative injuries and the greater degree of soft-tissue damage sustained by our ECI cohort may explain why, in these patients, cfDNA was positively associated with both hospital and ICU LOS at all 3 sampling time points. Indeed, when compared to isolated TBI patients, it is reasonable to think that in patients with polytrauma, longer hospital LOS is related to higher cfDNA levels based on the fact that they probably have a greater burden of injury. In addition to LOS, significantly higher cfDNA concentrations were measured in plasma samples obtained ≤1 hour and 4–12 hours post-injury from ECI patients who subsequently developed MODS, an observation that matches the results of previous prospective studies of hospitalized trauma patients [14, 49]. Of note, AUROC analyses revealed that a measurement of cfDNA in samples acquired within minutes of injury could moderately (AUROC 0.729, 95% CI 0.564–0.866) discriminate between ECI patients who did or did not develop MODS. Thus, taken together, these data suggest that an immediate assessment of cfDNA in patients that have sustained an ECI may help in the early identification of subjects at risk of poor outcome.

A number of groups have reported the presence of both nDNA and mtDNA in the circulation of trauma patients [4, 5, 26, 47, 50]. Derived from an organelle of bacterial origin, mtDNA shares many structural similarities with bacterial DNA, which includes a circular genome and the presence of unmethylated CpG motifs. Through activation of the pathogen recognition receptor toll-like receptor 9 and the DNA sensor cyclic GMP-AMP synthase, mtDNA triggers various pro-inflammatory responses [51, 52], which in the setting of traumatic injury has been shown to include the activation of neutrophils [3, 53, 54]. As such, mtDNA-driven inflammatory responses are thought to contribute to poor clinical outcomes post-injury. In line with this theory, significantly higher mtDNA levels have been detected in the post-hospital admission blood samples of non-survivors versus survivors of traumatic injury [4, 7], as well as in individuals who developed post-traumatic MODS [49, 55], ARDS [23] and a systemic inflammatory response [8, 26, 49]. In a previous study, in which we examined the origins of the cfDNA present within our patient blood samples, we found no difference in the concentration of mtDNA between patient samples and those of HCs [2]. However, we did observe a significant post-trauma elevation in the circulating levels of nDNA, suggesting that the associations we have reported in the current study between elevated cfDNA and poor clinical outcome are attributable to the actions of nDNA. On this note, in a recent prospective cohort study of 104 patients with severe blunt trauma, Stortz *et al.* found circulating concentrations of nDNA, but not mtDNA, were significantly higher ≤12 hours and 24 hours post-injury in patients who developed chronic critical illness when compared to those who experienced rapid recovery [47]. Furthermore, significantly elevated plasma concentrations of nDNA have been reported in non-survivors of major traumatic injury [4, 9] and in patients who develop post-traumatic MODS [14] and nosocomial infections [50]. Whilst appearing to be devoid of pro-inflammatory/immunogenic activity [18], nDNA and/or its associated proteins (e.g. histones) have been shown to trigger platelet activation [18, 20], inhibit fibrinolysis [56], perturb blood flow through capillary plexi [57] and induce endothelial/epithelial cell death [19, 21, 22]. Thus, these pro-thrombotic and cytotoxic properties of nDNA/histones may represent a potential mechanistic explanation for the associations that we and others have reported between raised circulating nDNA and poor clinical outcome post-injury [4, 9, 14].

In each of our patient cohorts, we detected, at all sampling time points, significantly elevated circulating concentrations of DNase-1. Expressed in a variety of tissues, DNase-1 localizes along the secretory pathway in Paneth cells of the small intestine and the hormone secreting cells of the pituitary gland [58, 59]. As blunt/penetrative trauma and TBI results in increased intestinal permeability and activation of the hypothalamic–pituitary–adrenal axis [60–62], the secretion/passive release of DNase-1 from these tissues could potentially explain the immediate and sustained elevation in the circulating levels of this endonuclease post trauma. The combination of raised DNase-1 antigen levels and cfDNA concentrations post trauma led us to investigate the effect of injury on the enzymatic activity of DNase. Confirming the findings of McIlroy *et al.*, who had previously reported impaired DNase-1 activity in a cohort of major orthopaedic blunt trauma patients [26], we found DNase activity was significantly reduced in serum samples obtained from ECI patients 4–12 hours and 48–72 hours post-injury. Furthermore, we have shown for the first time that serum DNase activity is significantly reduced in patients who have sustained an isolated TBI or TBI with an accompanying ECI. This impairment in enzymatic activity was detectable within minutes of injury and persisted for up to 72 hours post trauma.

Our observation of reduced DNase activity in the background of elevated protein levels led us to investigate whether a circulating inhibitor was contributing to the trauma-induced decline in DNase activity. Released by tissue injury, G-actin binds to and inhibits the nuclease activity of DNase-1 [32–34]. Screening of pre-hospital blood samples obtained from all patient groups revealed an immediate significant increase in the circulating concentration of G-actin. Whilst elevated, the differences we detected in G-actin levels between HCs and injured patients were not as marked as we had expected. We attribute this to the fact that our methodology is likely to have underestimated the exact concentration of G-actin present in the circulation post-injury, as the ELISA we used only measures free G-actin. Thus, the assay would not have detected G-actin bound to DNase-1 or VDBP. Given that actin–VDBP complexes are formed within minutes of injury [39], the total circulating pool of G-actin in the immediate aftermath of injury is likely to be much greater than that we have reported here.

Responsible for the clearance of actin from the circulation is the EASS, which is comprised of the plasma-residing proteins GSN and VDBP [35–37]. Mechanistically, GSN drives the depolymerization of F-actin into monomeric G-actin subunits that are subsequently sequestered by VDBP and cleared via a receptor-dependent mechanism in the liver, lung and/or spleen [63, 64]. As reported by others [39–44], we detected, across our 3 patient cohorts, significantly reduced concentrations of GSN and VDBP in pre- and/or post-hospital admission blood samples. When complexed to actin, the circulating half-lives of GSN and VDBP are markedly decreased [65]. Although not investigated in this study, previous analyses of hospital admission blood samples of polytrauma patients have revealed an increased frequency of VDBP–actin complexes [39]. Thus, we suggest that the deficiency we observed in VDBP and GSN reflects, at least in part, their consumption by circulating actin. In light of these data, we propose a post-injury scenario in which actin release from damaged tissue reduces plasma GSN and VDBP levels. This dysregulation in the EASS results in elevated concentrations of circulating G-actin, the subsequent binding of which to DNase-1 significantly reduces its enzymatic activity [32–34]. Impaired DNase-1–mediated clearance of cfDNA, combined with the release of DNA from damaged tissue and/or activated immune cells following both the initial act of trauma and subsequent surgical interventions, results in an immediate and persistent elevation in circulating cfDNA [5]. Through activation of the clotting cascade [18, 20], promotion of endothelial dysfunction [19, 21, 22] and reduction of capillary blood flow [57], raised cfDNA may contribute to the development of such secondary complications as MODS and ARDS that would result in an increased ICU/hospital LOS.

In rodent models of haemorrhagic shock and sepsis, clearance of circulating DNA via systemic administration of DNase-1 has been shown to protect against organ damage and promote host survival [66, 67]. As human-based trauma studies have shown, raised cfDNA levels are associated with the development of MODS [8, 14, 24], multiple organ failure [14] and an increased risk of mortality [4, 7, 9, 10, 12, 13], could DNase-1 treatment reduce the incidence of secondary complications amongst hospitalized trauma patients? Although feasible, this approach is faced with a number of obstacles. For instance, not only does the timing of DNase-1 treatment appear to be critical for its success [67], but, based on the data presented in this manuscript, exogenous DNase-1 would be administered into an environment rich in circulating G-actin. Combined with the post-trauma dysregulation in the EASS that persists in the days and weeks following injury [39–44], raised concentrations of G-actin would promote the formation of G-actin–DNase complexes, an association that negatively impacts upon the activity of DNase-1 [32–34]. Interestingly, in a small pilot study of polytrauma patients, we measured significantly increased GSN concentrations and DNase activity in blood samples acquired from individuals that had received fresh frozen plasma (FFP) prior to hospital admission [68]. Although the underlying mechanism was not directly demonstrated, we speculated that GSN-mediated scavenging of circulating actin was responsible for this FFP-induced enhancement in DNase activity [68]. Recently, a multicentre phase 3 clinical trial demonstrated that pre-hospital administration of thawed plasma to trauma patients at risk of haemorrhagic shock was safe and associated with a reduced risk of mortality when compared to standard care [69]. As we have provided evidence of elevated cfDNA levels and reduced DNase activity within minutes of injury, a large-scale pre-hospital based trial of trauma patients that investigates whether administration of FFP or recombinant GSN enhances DNase-mediated clearance of cfDNA, via restoration of the EASS, would build upon our previous pilot study [68]. This would help determine whether increasing DNase activity is a potential therapeutic strategy by which to improve the clinical outcomes of severely injured patients.

In addition to targeting DNase, direct scavenging of DNA is currently being investigated as a means of preventing the onset of secondary complications associated with raised cfDNA. In a rodent model of combined tissue injury and haemorrhagic shock, clearance of circulating DNA via systemic administration of a nucleic acid scavenging polymer was found to attenuate pro-inflammatory responses and protect against organ injury [55]. Highlighting how such a strategy could translate into the treatment of trauma patients, Gocho *et al.* recently demonstrated that direct haemoperfusion through filters containing immobilized polymyxin B significantly reduced circulating DNA levels in a cohort of septic patients, an approach that the authors suggested could be an effective means by which to reduce unwarranted inflammation and thrombogenesis [70].

Whilst in this article we have focused upon how trauma-induced alterations in the circulating concentrations of G-actin and the EASS may, via their regulation of DNase-1 activity, contribute to the post-injury persistence in circulating cfDNA, a review of the literature suggests that these changes may have additional implications for the host. For example, VDBP and GSN have been shown to: (1) augment C5a-induced chemotaxis of monocytes and neutrophils [71, 72], (2) increase both the phagocytic and microbicidal activity of alveolar macrophages [73] and (3) induce nitric oxide synthesis [73]. Furthermore, GSN has been proposed to exhibit immune regulatory properties. Referred to as the ‘GSN mediator buffer hypothesis’, this plasma protein has been shown to bind to and inhibit the agonistic activity of a range of inflammatory mediators that includes lysophosphatidic acid [74], platelet-activating factor [74], lipopolysaccharide [75] and lipoteichoic acid [76]. Based in part on data from animal models of infection/injury that have demonstrated reduced inflammatory complications and increased survival following GSN administration [77–79], it is believed that GSN helps prevent excessive systemic inflammatory responses. Thus, depletion of GSN and VDBP may be a contributory factor in both the impaired immune responses and elevated systemic inflammation observed in traumatically injured patients. Interestingly, this scenario may be exacerbated by the trauma-induced elevation in circulating actin, a protein that is both immune suppressive, as demonstrated by its ability to impair macrophage bacterial defences [80] and immune stimulatory, with its recognition by cross-presenting dendritic cells potentially contributing to the activation of CD8^+^ T cells and the induction of pro-inflammatory immune responses [81, 82]. Thus, via their ability to modulate host immune responses, the consequences of a dysregulated EASS and elevated levels of circulating actin may extend beyond a post-trauma accumulation of cfDNA.

## Conclusions

We have demonstrated a very rapid post-injury elevation in circulating cfDNA levels and a reduction in DNase activity in patients with both extracranial injuries and TBI. This impairment in endonuclease activity occurred secondary to reduced levels of the actin scavengers GSN and VDBP and elevated levels of G-actin. Impaired DNase activity offers a potential common mechanistic explanation for the persistently elevated concentrations of cfDNA associated with the development of secondary complications and/or death. Moreover, restoration of DNase-1 activity, which can be achieved via administration of FFP or GSN, may represent a therapeutic strategy to improve the clinical outcomes of hospitalized trauma patients.

## Data Availability

All data upon which the conclusions of the paper rely are presented in the main manuscript.

## Abbreviations

ARDS: acute respiratory distress syndrome; AUROC: area under the receiver operating characteristics; cfDNA: cell-free deoxyribonucleic acid; DNase: Deoxyribonuclease; DNase-1: deoxyribonuclease-1; EASS: extracellular actin scavenger system; ECI: extracranial injury; ELISA: enzyme-linked immunosorbent assay; F-actin: filamentous actin; FFP: fresh frozen plasma; G-actin: monomeric globular actin; GCS: Glasgow Coma Scale; GSN: gelsolin; HCs: healthy controls; ICU: intensive care unit; ISS: Injury Severity Score; LDH: lactate dehydrogenase; LOS: length of stay; MODS: multiple organ dysfunction syndrome; mtDNA: mitochondrial-derived DNA; nDNA: nuclear-derived DNA; NETs: neutrophil extracellular traps; NHS: National Health Service; NISS: New Injury Severity Score; PFP: platelet-free plasma; RT: room temperature; SOFA: Sequential Organ Failure Assessment; TBI: traumatic brain injury; TBI^+^: traumatic brain injury with accompanying extracranial injury; VDBP: vitamin D binding protein.
